# Dietary Carotenoids and Non-Alcoholic Fatty Liver Disease among US Adults, NHANES 2003–2014

**DOI:** 10.3390/nu11051101

**Published:** 2019-05-17

**Authors:** Krista Christensen, Thomas Lawler, Julie Mares

**Affiliations:** 1 Department of Ophthalmology and Visual Sciences, University of Wisconsin, 610 N. Walnut Street, 1069 WARF Building, Madison, WI 53726, USA; jmarespe@wisc.edu; 2 Department of Nutritional Sciences, University of Wisconsin, 1415 Linden Drive, Madison, WI 53706, USA; tlawler2@wisc.edu

**Keywords:** nutrition, chronic disease, lutein, zeaxanthin, lycopene, beta-carotene, alpha-carotene, beta-cryptoxanthin

## Abstract

Non-alcoholic fatty liver disease (NAFLD) is highly prevalent worldwide. Oxidative stress is thought to be a major mechanism, and previous epidemiological studies found higher serum levels of antioxidant carotenoids were associated with reduced risk for development and progression of NAFLD. The objective of this analysis is to examine cross-sectional associations between dietary and serum levels of carotenoids in relation to NAFLD among a nationally representative sample of US adults. We used data from the 2003–2014 National Health and Nutrition Examination Survey (NHANES). Dietary carotenoid intake was estimated from a 24-hour recall, while serum carotenoids were measured from 2003 to 2006. The NAFLD status was determined based upon US Fatty Liver Index (FLI) value ≥30. Regression models were used to estimate associations between carotenoids and NAFLD by controlling for covariates and adjusting for survey design variables. Overall, 33% of participants were classified as having NAFLD. Intake of all carotenoids, with the exception of lycopene, was lower among those with NAFLD. This association was significant for the highest quartiles of intake of α-carotene, β-carotene, β-cryptoxanthin, and lutein/zeaxanthin. For serum measures, the highest level of all carotenoids was associated with significantly reduced odds of NAFLD. In conclusion, higher intake and serum levels of most carotenoids were associated with lower odds of having NAFLD. Identification of such modifiable lifestyle factors provide an opportunity to limit or prevent the disease and its progression.

## 1. Introduction

Non-alcoholic fatty liver disease (NAFLD) is highly prevalent worldwide, and is the most common liver disease in the United States with an estimated prevalence of 30% among adults [1], and 3% to 12% among children [2]. NAFLD is conceptualized as the hepatic manifestation of the metabolic syndrome, and comprises a range of conditions across the clinical spectrum. The American Association for the Study of Liver Diseases defines NAFLD as presence of both “*evidence of hepatic steatosis (HS), either by imaging or histology*” and “*lack of secondary causes of hepatic fat accumulation such as significant alcohol consumption, long-term use of a steatogenic medication, or monogenic hereditary disorders*” [3]. Hepatic steatosis increases risk for progression to non-alcoholic steatohepatitis (NASH), which is characterized by activation of hepatic macrophages, infiltration of inflammatory immune cells, hepatocyte ballooning, and cell death. NASH, in turn, drastically increases risk for fibrosis, cirrhosis leading to liver failure, and hepatocellular carcinoma [3,4]. Since there is currently no accepted pharmaceutical or surgical treatment for NAFLD [5], lifestyle modifications such as dietary changes are typically recommended to prevent the development of NAFLD, and to mitigate severity once disease has developed.

NAFLD is thought to develop through a series of ‘hits’ or stressors to the liver (‘multiple hit’ hypothesis). Although the pathophysiology of NAFLD is complex, abdominal adiposity, aberrant lipid metabolism, insulin resistance, oxidative stress, and inflammation likely contribute to NAFLD by overlapping and mutually reinforcing pathways. The proximal cause of NAFLD is accumulation of fat in liver cells (hepatocytes) combined with oxidative stress and other insults to the liver. Hence, antioxidant activity by carotenoids may reduce risk, severity, or progression of this disease. A growing body of epidemiological research indicates that higher levels of carotenoids in serum are associated with reduced risk for NAFLD or for disease progression [6,7,8,9,10,11]. Previous studies found increased risk for NAFLD and NASH with lower levels of serum β-carotene, α-carotene, β-cryptoxanthin, and lycopene [7,10,12], and lower total serum carotenoids [6]. Higher levels of both specific and total serum carotenoids were prospectively associated with improvements in NAFLD as assessed by abdominal ultrasonography in a cohort of Chinese adults [11]. However, there is little evidence for association with dietary intake of carotenoids, and no identified trials of carotenoid interventions to treat NAFLD or slow its progression.

Despite the incomplete body of evidence from epidemiological studies, there are animal studies to support the association of carotenoid exposure and risk of NAFLD. Multiple rodent studies demonstrate that both lycopene [13,14,15,16] and lutein [17] can slow the rate of hepatic lipid accumulation in mice fed a high-fat diet. Proposed mechanisms for this effect include activation of SIRT1, which is a master regulator of mitochondrial biogenesis and lipid oxidation, and improvement in insulin sensitivity. Lycopene feeding also reduced markers of liver damage including ALT and AST [14,16], and attenuated circulating levels of pro-inflammatory cytokine TNF-α [14], implicated in NAFLD progression. The antioxidant capacity of carotenoids may directly prevent liver damage and progression of hepatic steatosis to NASH, by mitigating the injurious effects of oxidative stress in hepatocytes. Likewise, carotenoids may attenuate pro-inflammatory signaling through transcription factor NFKβ [18], and inhibit activation of macrophages to an M1 phenotype (characteristic of NAFLD progression) [19]. A recent review concluded that carotenoids may confer protection via multiple pathways, including decreased hepatic levels of cholesterol, glucose, MDA (malondialdehyde, marker of lipid peroxidation), TNF-α, and NFKβ binding activity [20]. 

In this analysis, we examine cross-sectional associations between dietary and serum carotenoids in relation to NAFLD-related outcomes among a nationally representative sample of US adults. We hypothesize that greater exposure to carotenoids in diet and serum will be associated with lower odds of NAFLD, as assessed when utilizing the US Fatty Liver Index (FLI). 

## 2. Materials and Methods

### 2.1. Design 

The National Health and Nutrition Examination Survey (NHANES) is a cross-sectional survey designed to provide a representative sample of the US non-institutionalized civilian population [21]. This work is not considered human subjects research since it relies on free, publicly available datasets only, and is, thus, not subject to IRB review.

For these analyses, we used information on dietary (2003–2014) and serum (2003–2006) carotenoids, and factors needed to construct the US FLI (race/ethnicity, age, gammaglutamyl transferase level, waist circumference, insulin level, and glucose level). Participants were excluded for the following reasons: missing information needed to calculate US FLI (e.g., non-participation in the fasting sample) or on carotenoid dietary intake, age less than 20 years, presence of hepatitis B or C antibody, missing or elevated alcohol intake (≥10 g or 1 drink/day for women or 20 g or 2 drinks/day for men), and self-reported liver disease.

### 2.2. Carotenoids in Diet and Supplements and in Serum

NHANES participants provide detailed dietary intake information for up to two 24-h periods. These recalls are used to estimate intakes of energy, nutrients, and other food components. The first dietary recall is collected in-person during the NHANES visit, while the second recall is collected by telephone 3 to 10 days later. For these analyses, total estimated dietary carotenoid intake (micrograms, µg) was averaged over the two recall periods (if only the first day was available, that value was used). For the NHANES cycles used, information was available on α-carotene, β-carotene, β-cryptoxanthin, lycopene, and lutein/zeaxanthin (combined). While the main focus of this analysis is on dietary intake, participants are also queried about supplement use for the same two 24-h periods with respect to carotenoids and supplement intake of lycopene and lutein/zeaxanthin were available for later NHANES cycles (2007 onward). For participants reporting supplement use, total intake of lycopene and of lutein/zeaxanthin was calculated as the sum of dietary and supplement intake for sensitivity analyses.

For two of the NHANES cycles in this analysis (2003/2004, 2005/2006), serum measurements of carotenoids were also available. In these two cycles, participants aged 6 years and older provided serum samples for measurement of six carotenoids (α-carotene, trans-β-carotene, cis β-carotene, β-cryptoxanthin, combined lutein/zeaxanthin, trans-lycopene, and total lycopene) using high performance liquid chromatography (HPLC). We evaluated serum levels of carotenoids in relation to NAFLD outcomes as a sub-analysis.

### 2.3. NAFLD-Related Outcomes

Outcome status is based upon the US Fatty Liver Index (FLI), as described in Ruhl et al. [22]. The FLI was developed using NHANES III data and evaluated against hepatic steatosis, as diagnosed by an abdominal ultrasound. A cutoff of 30 was used to define NAFLD as suggested by the authors.

### 2.4. Statistical Analysis

All data analysis was performed using SAS/STAT software (Version 9.4, SAS Institute Inc., Cary, NC, USA). Regression models were used to identify associations between carotenoid intake and NAFLD-related outcomes, controlling for potential confounders. The primary models included adjustment for survey design variables, and for survey cycle, sex, and age in years. To evaluate whether the effects of carotenoid intake might be attributable more generally to a healthy diet, we also looked at models additionally adjusted for the healthy eating index (HEI) 2015 score. This score was only available for 2005 onwards. As a sensitivity analysis, we evaluated regression models including primary adjustment variables, as well as poverty income ratio (PIR) and educational attainment. These additional factors may be related to both carotenoid intake and risk for NAFLD, but it is likely that their association with NAFLD is mediated through diet. Serum cotinine as a proxy for smoke exposure was evaluated, but was not associated with carotenoid intake nor with an NAFLD status. To evaluate potential non-linear effects, carotenoid exposures were treated as both continuous and categorical (quartiles) variables. Statistically significant was defined as *p*-value < 0.05 for categorical variables and for a linear trend. 

Both logistic (binary outcome of NAFLD, SURVEYLOGISTIC procedure) and linear (continuous outcome of US FLI, SURVEYREG procedure) regression models were used to evaluate associations with carotenoid intake. All statistical analyses were adjusted for survey design and weighting variables. For the full dataset analysis (dietary intake of carotenoids), we created 12-year weights as one-sixth of the value of the fasting subsample MEC weight (WTSAF2YR * 1/6) since this represented the smallest subsample of the study population. For sensitivity analyses of total (diet and supplement) lycopene and lutein/zeaxanthin intake, eight-year weights were calculated as one fourth of the value (WTSAF2YR * 1/4). When including HEI 2015 score as an adjustment variable, we used 10-year weights calculated as one-fifth the value (WTSAF2YR * 1/5). For the sub-analysis of serum carotenoids, we created 4-year weights as one-half of the value (WTSAF2YR * 1/2), and for analysis of serum carotenoids including HEI 2015 score (2005–2006 serum data only), the original 2-year weights were used.

## 3. Results

Table 1 displays characteristics of the study population stratified by NAFLD status. About one-third (33%) were classified as having NAFLD based on a US FLI score. Not surprisingly, those with NAFLD tended to be older and were more often male, Mexican-American, or non-Hispanic white, and were more likely to be obese or have diabetes or hypertensive factors. 

Intake of all carotenoids with the exception of lycopene, was lower among those with NAFLD. The dietary intakes of the specific carotenoids did show correlations in some cases. When accounting for survey weighting, Pearson correlations were highest between intakes of α and β-carotene (ρ = 0.75), α-carotene, and lutein/zeaxanthin (ρ = 0.48), and β-carotene and lutein/zeaxanthin (ρ = 0.69). Correlations between other carotenoids were lower but significant. When looking at the NHANES cycles where serum carotenoids were measured, all were highly correlated with each other (*p* < 0.001). An especially high correlation was noted between serum α and β-carotene levels (ρ = 0.66), while other correlations were weaker (ρ = 0.19−0.45) but still highly significant. Dietary intake levels for each carotenoid were significantly associated with corresponding serum levels (*p* < 0.001 for all) although Pearson correlation coefficients were modest (ρ = 0.25−0.37), which may reflect an error in assessment of carotenoid intake, or inter-individual variation in carotenoid absorption.

Table 2 shows odd ratios (ORs, 95% confidence intervals [CIs]) for the presence of NAFLD as a binary outcome. Analogous results from modeling the US FLI score as a continuous outcome are shown in Supplementary Table S1. In both tables, results are presented treating carotenoid intake as a categorical variable (quartiles) since there was evidence of non-linearity in some cases. However, we do include a *p*-value for the trend calculated from models including the carotenoid as a continuous exposure, for reference. In adjusted logistic models, the highest quartiles of intake of α-carotene, β-carotene, β-cryptoxanthin, and lutein/zeaxanthin were all associated with lower odds of NAFLD compared to the lowest quartile of intake. Associations were attenuated when including the HEI 2015 score in the model, but the direction of association was unchanged and remained significant for β-carotene and lutein/zeaxanthin. In sensitivity analyses including education and income, results were largely similar but somewhat attenuated. Similarly, significant associations in linear models for US FLI as a continuous outcome were seen for the highest quartiles of intake of each carotenoid with the exception of lycopene. In logistic regression models, results were attenuated when including the HEI 2015 score (or education and income) but unchanged with respect to the direction of association.

Table 3 displays results from logistic regression modeling of serum carotenoid exposures and the NAFLD status as a binary outcome, while analogous results for US FLI as a continuous outcome are shown in Supplementary Table S2. As shown above for dietary intakes, serum carotenoids were treated as categorical exposures, with p-value for the trend calculated from models including serum carotenoids as a continuous variable. For serum carotenoid levels, a dose-response pattern was more apparent (compared with dietary intake exposures), with most of the *p*-values for the trend being statistically significant. In the fully adjusted model, the highest quartile was associated with significantly reduced risk for NAFLD among all carotenoids except lycopene. Statistically significant inverse associations were observed for Quartiles 2–4 of serum β-carotene, and Quartiles 3 and 4 of serum α-carotene, β-cryptoxanthin, and lutein/zeaxanthin. In general, estimates were largely unaffected by adjusting the HEI 2015 score, or for income and education. Similar results were seen in fully adjusted linear models for US FLI as a continuous outcome, but the highest quartile of serum lycopene was associated with lower US FLI in the fully adjusted model (see Supplementary Table S2).

## 4. Discussion

In this nationally representative sample of the US adult population, higher intake of certain carotenoids, and higher concentrations of carotenoids in the serum were associated with lower odds of having NAFLD, and with a better score of the US Fatty Liver Index. This is very promising given that there are no currently accepted therapeutic interventions for NAFLD. The dietary modification to increase carotenoid intake represents a possible route for prevention and amelioration of a common chronic health condition. 

These findings build on a body of evidence suggesting relationships between carotenoid status to lipid metabolism and obesity, and are consistent with other epidemiological studies that demonstrate a prospective association between serum carotenoid levels and NAFLD [6,7,11], or between serum carotenoids and serum ALT [8,9]. These previous studies included a variety of designs. In a cross-sectional prevalence study (similar to the design of the NHANES analysis reported here), Cao et al. demonstrated that individuals with the highest serum levels of α and β-carotene, β-cryptoxanthin, lycopene, and lutein/zeaxanthin, had significantly reduced risk (ORs 0.32–0.62) for NAFLD, as diagnosed using abdominal ultrasonography [6]. A prospective study from the same group revealed that serum carotenoids were approximately 9% to 30% higher in individuals whose NAFLD improved compared to those who experienced disease progression [11]. Lastly, a case-control study demonstrated that serum lutein, zeaxanthin, lycopene, and α and β-carotene levels were 30% to 60% lower in NASH cases compared to controls [7]. Based on our knowledge, the present study is the first to report a statistically significant inverse association between dietary carotenoid intake and both risk of NAFLD and score on the US FLI, which is consistent with reduced risk for NAFLD. Our results are supported by animal studies in which the administration of carotenoids (including lycopene and lutein) attenuated the hepatic accumulation of lipids on a high-fat diet, as well as increased expression of SIRT1, which is a key regulator of fatty acid oxidation [15,17]. Higher carotenoid intake may reduce risk for NAFLD, and, in particular, the progression of simple hepatic steatosis to NASH, through several different pathways, including the attenuation of oxidative stress in the liver, with downstream effects on secretion of pro-inflammatory cytokines by hepatic macrophages, immune infiltration, and insulin sensitivity [20].

Many of the carotenoid measures were correlated with each other, which can make it difficult to distinguish individual effects (for example, correlations between intakes of α and β-carotene, and each of these with lutein/zeaxanthin, were between 0.78 to 0.94). For the logistic model (NAFLD as a binary outcome), when comparing the fully adjusted model with one carotenoid included at a time versus all included in the model, effect estimates were generally similar but attenuated (and confidence intervals increased in width). We investigated the possibility that beneficial associations of higher carotenoid intake are due to benefits of eating foods high in carotenoids (e.g., fruits and vegetables). Inclusion of the healthy eating index 2015 score did attenuate—but did not eliminate—associations with dietary carotenoids. Conversely, associations for serum carotenoids were largely unaffected by adjusting the healthy eating index score. It is likely that some of the protective association is due to healthier eating patterns. In addition, healthy diet patterns may enhance absorption of carotenoids from the diet. However, some effect may be due to carotenoid mechanisms specifically. This would be consistent with the observation that inverse associations were generally stronger and more consistent for serum carotenoids compared with dietary intake, which may reflect heterogeneity in the absorption of carotenoids into the bloodstream and greater measurement error or misclassification in the self-reported dietary data [23].

Limitations of this analysis included the use of self-reported dietary data, which may be due to a greater measurement error. The NAFLD status was inferred based upon a previously validated index, but is not a perfect proxy for NAFLD diagnosis based upon liver biopsy or other more invasive methods. However, evaluation of the FLI in the NHANES has shown good sensitivity, with the area under the receiver operator curve reported to be 78% [22], which suggests less potential for the outcome misclassification error. Furthermore, use of this index with the NHANES population made it impossible to adjust for potentially important covariates including body composition and markers of insulin sensitivity. Since the FLI is a surrogate marker for NAFLD based on markers of metabolic health, it is not clear whether carotenoid exposure is correlated with NAFLD independently of these markers. Another limitation is that the dietary analysis does not include an estimation of intake for certain carotenoids—such as astaxanthin and fucoxanthin—which may also have associations with NAFLD. Strengths include the use of multiple years of data from a large and nationally representative sample, consideration of multiple potential confounders, and examination of multiple carotenoids singly and in combination.

## 5. Conclusions

We found that higher intake and serum levels of certain carotenoids were cross-sectionally associated with lower odds of NAFLD. These findings are promising given the limited therapeutic options for treating NAFLD, in that identification of modifiable lifestyle factors provide an opportunity to limit or prevent the disease and its progression.

## Figures and Tables

**Table 1:** Characteristics of NHANES participants, 2003–2014, by the NAFLD status.

Group	No NAFLD		NAFLD		*p*-Value * for Difference between Those With and Without NAFLD
	Weighted *n* (SD)	Weighted Percent (SE)	Weighted *n* (SD)	Weighted Percent (SE)	
Total	61,456,846 (2,218,754)	100 (0)	30,295,373 (1,301,957)	100 (0)	
Survey Cycle					0.02
2003–2004	10,566,907 (1,252,551)	17.2 (1.8)	4,397,048 (603,680)	14.5 (1.8)	
2005–2006	9,983,650 (701,413)	16.2 (1.1)	4,560,556 (428,466)	15.1 (1.3)	
2007–2008	10,630,944 (843,068)	17.3 (1.3)	5,188,078 (602,156)	17.1 (1.8)	
2009–2010	9,483,325 (665,022)	15.4 (1.1)	5,996,470 (478,479)	19.8 (1.5)	
2011–2012	10,439,332 (1,024,219)	17.0 (1.5)	4,980,308 (513,756)	16.4 (1.6)	
2013–2014	10,352,685 (812,379)	16.8 (1.2)	5,172,910 (539,998)	17.1 (1.6)	
Age Group (years)					<0.0001
20–39	20,935,959 (928,501)	35.8 (1.2)	6,195,884 (420,329)	21.1 (1.2)	
40–59	20,750,665 (1,067,685)	35.5 (1.2)	11,253,296 (747,129)	38.4 (1.6)	
60–79	13,098,637 (712,268)	22.4 (0.8)	10,320,193 (568,536)	35.2 (1.5)	
≥80	3,682,338 (262,507)	6.3 (0.4)	1,547,004 (175,185)	5.3 (0.6)	
Sex					<0.0001
Male	26,036,711 (1,090,038)	42.4 (0.9)	16,577,926 (822,807)	54.7 (1.2)	
Female	35,420,134 (1,373,568)	57.6 (0.9)	13,717,446 (675,055)	45.3 (1.2)	
Race/Ethnicity					<0.0001
Mexican American	4,040,549 (353,152)	6.6 (0.7)	3,214,449 (336,258)	10.6 (1.3)	
Other Hispanic	2,502,869 (294,586)	4.1 (0.5)	1,169,495 (146,235)	3.9 (0.5)	
Non-Hispanic White	42,084,359 (2,357,817)	68.5 (1.6)	22,580,348 (1,364,103)	74.5 (1.8)	
Non-Hispanic Black	8,406,747 (589,390)	13.7 (1.1)	1,986,698 (189,877)	6.6 (0.7)	
Other/Multiracial	4,422,319 (386,390)	7.2 (0.6)	1,344,381 (187,613)	4.4 (0.6)	
BMI Category					<0.0001
Underweight (<18.5)	1,267,523 (190,732)	2.1 (0.3)	11,483 (11,483)	0 (0)	
Normal (18.5–24.9)	23,267,000 (1,199,452)	37.9 (1.1)	1,067,429 (166,431)	3.5 (0.5)	
Overweight (25–29.9)	24,039,650 (975,583)	39.1 (1)	6,865,896 (515,913)	22.7 (1.3)	
Obese (≥30)	12,882,671 (625,090)	21.0 (0.8)	22,350,563 (1,000,989)	73.8 (1.4)	
Smoking Status					<0.0001
Never	39,936,202 (1,411,228)	65 (1)	17,112,945 (831,979)	56.5 (1.5)	
Current	8,772,599 (631,562)	14.3 (0.8)	3,937,101 (329,377)	13 (1)	
Former	12,734,901 (761,119)	20.7 (0.9)	9,242,180 (599,355)	30.5 (1.2)	
Missing	13,143 (7800)	0 (0)	3146 (3146)	0 (0)	
Education Level					<0.0001
Less than HS	996,9649 (566,262)	16.2 (0.9)	6,847,959 (414,383)	22.6 (1.3)	
HS or some college	31,596,068 (1,513,799)	51.4 (1.3)	17,238,418 (1,037,220)	56.9 (1.7)	
College or more	19875667 (1,131,428)	32.3 (1.4)	6,208,995 (464,059)	20.5 (1.4)	
Missing	15,459 (9780)	0 (0)	0 (0)	0 (0)	
Diabetes (doctor’s diagnosis)					<0.0001
No	57,768,923 (2,130,004)	94 (0.5)	24,365,926 (1,187,983)	80.4 (1.3)	
Yes	3687,922 (301,760)	6 (0.5)	5,929,446 (413,180)	19.6 (1.3)	
Diabetes (plasma fasting glucose ≥127 mg/dL)					<0.0001
No	58,992,373 (2,196,856)	96 (0.4)	23,910,800 (1,125,079)	78.9 (1.2)	
Yes	2,464,472 (221,631)	4 (0.4)	6,384,573 (417,802)	21.1 (1.2)	
Diabetes (either criteria)					<0.0001
No	56,999,582 (2,117,147)	92.7 (0.5)	22,113,112 (1,076,534)	73 (1.3)	
Yes	4,457,263 (331,259)	7.3 (0.5)	8,182,260 (487,535)	27 (1.3)	
High blood pressure at exam (systolic ≥130 and/or diastolic ≥85 mm Hg)					<0.0001
No	44,620,732 (1,833,006)	74.5 (0.9)	17,861,138 (883,010)	61.1 (1.4)	
Yes	15,263,826 (689,875)	25.5 (0.9)	11,373,199 (622,176)	38.9 (1.4)	
	**Mean (SE)**		**Mean (SE)**		
Age (years)	48.28 (0.4)		53.77 (0.47)		<0.0001
Family Income (poverty income ratio, PIR)	3.02 (0.04)		2.83 (0.06)		0.001
Body Mass Index (kg/m^2^)	26.59 (0.1)		34.66 (0.23)		<0.0001
Waist Circumference (cm)	92.81 (0.25)		114.7 (0.47)		<0.0001
*Dietary Measures*					
Total Energy (kcal/day)	2018.42 (17.58)		2070.52 (23.51)		0.05
Dietary α-carotene (mcg)	489.85 (51.53)		387.73 (25.77)		0.16
Dietary β-carotene (mcg)	2547.38 (170.97)		1933.4 (70.65)		0.0003
Dietary β-cryptoxanthin (mcg)	110.22 (4.6)		93.48 (5.15)		0.05
Dietary lycopene (mcg)	5372.32 (190.38)		5624.4 (228.24)		0.37
Dietary lutein/zeaxanthin (mcg)	1748.17 (109.42)		1275.8 (49.83)		<0.0001
*Laboratory Measures*					
Insulin (pmol/L)	47.3 (0.62)		137.44 (3.38)		<0.0001
Glucose, plasma (mg/dL)	98.98 (0.43)		119.59 (1.17)		<0.0001
Cholesterol (mg/dL)	196.21 (1)		194.76 (1.33)		0.35
Triglycerides (mg/dL)	107.73 (2.15)		175.63 (4.18)		<0.0001
LDL-cholesterol (mg/dL)	116.69 (0.78)		115.08 (1.13)		0.24
Direct HDL-Cholesterol (mg/dL)	56.4 (0.38)		45.29 (0.34)		<0.0001
Gamma glutamyl transferase (U/L)	18.58 (0.26)		35.82 (0.85)		<0.0001
US Fatty Liver Index	12.39 (0.17)		52.8 (0.52)		<0.0001
*Serum Measures (2003–2006 only)*					
Serum α-carotene (µg/dL)	5.03 (0.28)		2.96 (0.14)		<0.0001
Serum β-carotene (µg/dL)	23.25 (1.14)		12.96 (0.6)		<0.0001
Serum β-cryptoxanthin (µg/dL)	10.32 (0.39)		7.6 (0.29)		<0.0001
Serum lycopene (µg/dL)	43.8 (0.72)		40.37 (1.07)		0.02
Serum lutein/zeaxanthin (µg/dL)	16.95 (0.42)		14.17 (0.34)		<0.0001

* Based on logistic regression with each independent variable considered separately. All analyses are adjusted for survey design variables.

**Table 2:** Odds ratios * (95% confidence interval) for association between the quartile of carotenoid intake, relative to Quartile 1, and presence of NAFLD. The median (range) for each quartile of carotenoid intake is also displayed (µg/day).

	Quartile 1	Quartile 2	Quartile 3	Quartile 4	*p*-Value for Trend
α-carotene					
Median (Range)	10.0 (0–27)	49.8 (27–95.5)	217.4 (96–477)	1007.4 (477.5–72,037)	
Model 1	Referent	0.99 (0.84, 1.16)	0.95 (0.79, 1.15)	**0.75 (0.63, 0.89)**	0.11
Model 2	Referent	**1.23 (1.04, 1.46)**	1.19 (0.98, 1.44)	0.99 (0.81, 1.21)	0.37
β-carotene					
Median (Range)	248.8 (0–435)	682.6 (435.5–1092.5)	1738.8 (1094–2726.5)	4691.2 (2728–246,122)	
Model 1	Referent	0.99 (0.82, 1.20)	**0.82 (0.70, 0.97)**	**0.63 (0.52, 0.76)**	**<0.001**
Model 2	Referent	1.04 (0.84, 1.28)	0.92 (0.76, 1.12)	**0.75 (0.60, 0.94)**	**0.02**
β-cryptoxanthin					
Median (Range)	5.7 (0–14.5)	26.3 (15–47)	76.6 (47.5–124)	229.5 (124.5–6088.5)	
Model 1	Referent	0.86 (0.72, 1.03)	0.82 (0.64, 1.04)	**0.64 (0.52, 0.80)**	**0.04**
Model 2	Referent	0.91 (0.74, 1.11)	0.97 (0.73, 1.28)	0.84 (0.65, 1.09)	0.50
Lycopene (Diet)					
Median (Range)	4.1 (0–564.5)	1273.5 (566.5–2233)	3792.1 (2234.5–6417.5)	12110 (6419–108,852)	
Model 1	Referent	0.94 (0.78, 1.14)	0.89 (0.74, 1.09)	1.12 (0.91, 1.37)	0.29
Model 2	Referent	0.99 (0.80, 1.23)	0.93 (0.75, 1.16)	1.07 (0.84, 1.35)	0.97
Lutein/Zeaxanthin (Diet)					
Median (Range)	277.0 (0–430.5)	600.2 (431–786.5)	1050.7 (787–1506)	2570.3 (1506.5–146,912)	
Model 1	Referent	0.94 (0.75, 1.17)	0.88 (0.73, 1.06)	**0.63 (0.51, 0.77)**	**<0.001**
Model 2	Referent	1.02 (0.81, 1.28)	1.02 (0.83, 1.26)	**0.78 (0.63, 0.96)**	**<0.001**
Total Lycopene (Diet and Supplement)					
Median (Range)	35.9 (0–590.5)	1313.8 (591–2276)	3838.2 (2280–6451.5)	12,203 (6455–108,852)	
Model 1	Referent	0.97 (0.81, 1.17)	0.92 (0.76, 1.1)	1.12 (0.92, 1.37)	0.42
Model 2	Referent	1.04 (0.86, 1.27)	0.96 (0.79, 1.18)	1.08 (0.86, 1.35)	0.72
Total Lutein/Zeaxanthin (Diet and Supplement)					
Median (Range)	281.6 (0–444.5)	627.6 (445–816.5)	1096.9 (817–1556)	2751.3 (1556.5–161,912)	
Model 1	Referent	0.92 (0.75, 1.14)	0.85 (0.71, 1.02)	**0.59 (0.48, 0.73)**	**<0.001**
Model 2	Referent	1.02 (0.82, 1.28)	1.01 (0.81, 1.25)	**0.73 (0.59, 0.91)**	**0.002**

* Model 1 is adjusted for age, sex, and the survey cycle year. Model 2 is adjusted for age, sex, and survey year, along with HEI 2015 score. All analyses are adjusted for survey design variables. Bold text indicates statistically significant associations.

**Table 3:** Logistic regression results (odds ratio (95% confidence interval)) for association between the quartile of serum carotenoids, relative to Quartile 1, and presence of NAFLD. The median (range) for each quartile of serum concentration is also displayed (µg/dL).

	Quartile 1	Quartile 2	Quartile 3	Quartile 4	*p*-Value for Trend
α-carotene					
Median (Range)	1.0 (0.2–1.5)	2.1 (1.6–3.0)	4.0 (3.0–5.4)	8.6 (5.4–96.5)	
Model 1	Referent	0.81 (0.62, 1.06)	**0.48 (0.33, 0.69)**	**0.33 (0.22, 0.5)**	**<0.001**
Model 2	Referent	0.7 (0.43, 1.13)	**0.35 (0.17, 0.71)**	**0.3 (0.15, 0.6)**	**<0.001**
β-carotene					
Median (Range)	6.0 (0.6–8.3)	10.5 (8.3–14.1)	18.2 (14.1–25.1)	36.1 (25.1–292.9)	
Model 1	Referent	**0.40 (0.28, 0.57)**	**0.31 (0.20, 0.46)**	**0.14 (0.09, 0.21)**	**<0.001**
Model 2	Referent	**0.28 (0.18, 0.45)**	**0.26 (0.14, 0.48)**	**0.13 (0.07, 0.23)**	**<0.001**
β-cryptoxanthin					
Median (Range)	3.5 (0.6–4.9)	6.4 (4.9–8.2)	10.3 (8.3–13.8)	19.4 (13.8–93.1)	
Model 1	Referent	0.73 (0.52, 1.03)	**0.49 (0.35, 0.68)**	**0.37 (0.26, 0.52)**	**<0.001**
Model 2	Referent	0.75 (0.44, 1.28)	**0.53 (0.31, 0.91)**	**0.45 (0.22, 0.92)**	**0.02**
Lycopene					
Median (Range)	19.2 (0.7–26.2)	32.6 (26.2–37.9)	43.5 (37.9–51.1)	64.0 (51.2–148.0)	
Model 1	Referent	0.91 (0.62, 1.32)	0.8 (0.53, 1.21)	**0.63 (0.41, 0.96)**	**0.03**
Model 2	Referent	0.99 (0.66, 1.49)	0.75 (0.4, 1.41)	0.68 (0.39, 1.21)	0.17
Lutein/Zeaxanthin					
Median (Range)	8.6 (2.4–10.9)	13.0 (10.6–15.2)	17.7 (15.2–27.0)	26.0 (20.7–113.1)	
Model 1	Referent	0.72 (0.51, 1.01)	**0.54 (0.36, 0.79)**	**0.33 (0.24, 0.47)**	**<0.001**
Model 2	Referent	0.78 (0.44, 1.38)	**0.53 (0.29, 0.97)**	**0.31 (0.2, 0.5)**	**<0.01**

Model 1 is adjusted for age, sex, and the survey cycle year. Model 2 is adjusted for age, sex, and survey year, along with the HEI 2015 score. All analyses are adjusted for survey design variables. Bold text indicates statistically significant associations.

## Supplementary Materials

The following are available online at https://www.mdpi.com/2072-6643/11/5/1101/s1. Table S1: Beta coefficients (standard error), *p*-values, for association between quartile of carotenoid intake, relative to quartile 1, and the US Fatty Liver Index. The median (range) for each quartile of carotenoid intake is also displayed (µg/day). Table S2: Beta coefficients (standard error), *p*-values, for association between quartile of serum carotenoids, relative to quartile 1, and the US Fatty Liver Index. The median (range) for each quartile of serum concentration is also displayed (µg/dL).
