# Supplementary material for: Dietary Carotenoids and Non-Alcoholic Fatty Liver Disease among US Adults, NHANES 2003–2014
Source: Nutrients. 2019 May 17;11(5):1101. doi: 10.3390/nu11051101 (PMC6566688; doi:10.3390/nu11051101)
Supplement: Supplementary file 1 [file nutrients-11-01101-s001.pdf]

Supplementary Table S1. Beta coefficients (standard error), p-values, for association between quartile of carotenoid intake, relative to quartile 1, and US Fatty Liver Index. The median (range) for each quartile of carotenoid intake is also displayed (μg/day).

	**Quartile 1**	**Quartile 2**	**Quartile 3**	**Quartile 4**	***p*-value for trend**
α-carotene					
Median (Range)	10.0 (0–27)	49.8 (27–95.5)	217.4 (96–477)	1007.4 (477.5–72,037)	
Model 1	Referent	0.34 (0.86), *p* = 0.69	−0.1 (0.98), *p* = 0.92	**−2.89 (0.9), *p*<0.01**	**<0.001**
Model 2	Referent	**2.67 (0.93), *p* = 0.01**	**2.5 (1.01), p = 0.02**	0.29 (1.05), *p* = 0.78	**<0.001**
β-carotene					
Median (Range)	248.8 (0–435)	682.6 (435.5–1092.5)	1738.8 (1094–2726.5)	4691.2 (2728–246122)	
Model 1	Referent	0.77 (0.98), *p* = 0.44	**−2.01 (0.81), *p* = 0.01**	**−4.58 (0.99), *p*<0.01**	**0.03**
Model 2	Referent	1.53 (1.03), *p* = 0.14	−0.22 (0.91), *p* = 0.81	**−2.33 (1.09), *p* = 0.04**	**0.01**
β-cryptoxanthin					
Median (Range)	5.7 (0–14.5)	26.3 (15–47)	76.6 (47.5–124)	229.5 (124.5–6088.5)	
Model 1	Referent	−1.65 (0.81), *p* = 0.05	−1.7 (1.11), *p* = 0.13	**−4.13 (1), *p*<0.01**	**0.02**
Model 2	Referent	−0.54 (0.87), *p* = 0.54	0.45 (1.25), *p* = 0.72	−1.13 (1.19), *p* = 0.35	0.69
Lycopene (Diet)					
Median (Range)	4.1 (0–564.5)	1273.5 (566.5–2233)	3792.1 (2234.5–6417.5)	12110 (6419–108852)	
Model 1	Referent	−1.15 (0.88), *p* = 0.19	−0.63 (1.02), *p* = 0.54	0.80 (1.03), *p* = 0.44	0.81
Model 2	Referent	−0.55 (0.94), *p* = 0.57	−0.27 (1.11), *p* = 0.81	0.51 (1.08), *p* = 0.64	0.86
Lutein/Zeaxanthin (Diet)					
Median (Range)	277.0 (0–430.5)	600.2 (431–786.5)	1050.7 (787–1506)	2570.3 (1506.5–146912)	
Model 1	Referent	−0.04 (1.02), *p* = 0.97	−1.12 (1.01), *p* = 0.27	**−5.07 (1.04), *p*<0.01**	**0.01**
Model 2	Referent	1.30 (1.08), *p* = 0.23	0.95 (1.12), *p* = 0.40	**−2.54 (1.1), *p* = 0.02**	**<0.001**
Total Lycopene (Diet and Supplement)					
Median (Range)	35.9 (0–590.5)	1313.8 (591–2276)	3838.2 (2280–6451.5)	12203 (6455–108852)	
Model 1	Referent	−0.63 (0.84), *p* = 0.45	−0.38 (0.96), *p* = 0.69	0.93 (1.01), *p* = 0.36	0.94
Model 2	Referent	0.22 (0.87), *p* = 0.8	0.06 (1.01), *p* = 0.95	0.73 (1.05), *p* = 0.49	0.57
Total Lutein/Zeaxanthin (Diet and Supplement)					
Median (Range)	281.6 (0–444.5)	627.6 (445–816.5)	1096.9 (817–1556)	2751.3 (1556.5–161912)	
Model 1	Referent	−0.58 (0.99), *p* = 0.56	−1.33 (1.01), *p* = 0.19	**−5.64 (1.06), *p*<0.01**	**0.01**
Model 2	Referent	0.78 (1.06), *p* = 0.46	0.7 (1.15), *p* = 0.54	**−3.2 (1.12), *p* = 0.01**	**<0.001**

*Model 1 is adjusted for age, sex, and survey cycle year. Model 2 is adjusted for age, sex and survey year, along with HEI 2015 score. Bold text indicates statistically significant associations.

Supplementary Table S2. Beta coefficients (standard error), p-values, for association between quartile of serum carotenoids, relative to quartile 1, and US Fatty Liver Index. The median (range) for each quartile of serum concentration is also displayed (μg/dL).

	Quartile 1	Quartile 2	Quartile 3	Quartile 4	p-value for trend
α-carotene					
Median (Range)	1.0 (0.2–1.5)	2.1 (1.6–3.0)	4.0 (3.0–5.4)	8.6 (5.4–96.5)	
Model 1	Referent	−2.82 (1.37), p = 0.05	**−8.30 (1.36), p<0.01**	**−12.16 (1.22), p<0.01**	**<0.001**
Model 2	Referent	−1.72 (2.54), P = 0.51	**−8.98 (2.57), p<0.01**	**−12.43 (2.28), P<0.01**	**<0.001**
β-carotene					
Median (Range)	6.0 (0.6–8.3)	10.5 (8.3–14.1)	18.2 (14.1–25.1)	36.1 (25.1–292.9)	
Model 1	Referent	**−8.53 (1.57), p<0.01**	**−12.3 (1.77), p<0.01**	**−19.29 (1.69), p<0.01**	**<0.001**
Model 2	Referent	**−10.11 (2.05), P<0.01**	**−13.06 (2.78), P<0.01**	**−20.72 (2.19), P<0.01**	**<0.001**
β-cryptoxanthin					
Median (Range)	3.5 (0.6–4.9)	6.4 (4.9–8.2)	10.3 (8.3–13.8)	19.4 (13.8–93.1)	
Model 1	Referent	−3.43 (1.64), p = 0.05	**−8.45 (1.15), p<0.01**	**−11.12 (1.31), p<0.01**	**<0.001**
Model 2	Referent	−2.22 (2.63), p = 0.41	**−7.78 (1.72), p<0.01**	**−9.46 (2.52), p<0.01**	**<0.001**
Lycopene					
Median (Range)	19.2 (0.7–26.2)	32.6 (26.2–37.9)	43.5 (37.9 – 51.1)	64.0 (51.2 – 148.0)	
Model 1	Referent	−0.98 (1.99), p = 0.63	−3.02 (2.42), p = 0.22	**−6.01 (1.79), p<0.01**	**<0.01**
Model 2	Referent	−1.3 (2.13), p = 0.55	−5.21 (3.25), p = 0.13	**−6.03 (2.37), p = 0.02**	**0.03**
Lutein/Zeaxanthin					
Median (Range)	8.6 (2.4–10.9)	13.0 (10.6–15.2)	17.7 (15.2–27.0)	26.0 (20.7–113.1)	
Model 1	Referent	**−4.46 (1.72), p = 0.01**	**−7.66 (1.59), p<0.01**	**−12.06 (1.56), p<0.01**	**< 0.001**
Model 2	Referent	−3.54 (2.73), p = 0.22	**−7.9 (2.98), P = 0.02**	**−12.28 (2.15), P<0.01**	**< 0.001**

*Model 1 is adjusted for age, sex, and survey cycle year. Model 2 is adjusted for age, sex and survey year, along with HEI 2015 score. Bold text indicates statistically significant associations.
